# Effects of Dual-Tasking on Stepping Strategy and Inter-Joint Coordination During Walking in Older Fallers and Non-Fallers

**DOI:** 10.1093/geroni/igaf055

**Published:** 2025-05-24

**Authors:** Ziwei Zeng, Cheuk-yin Ho, Junhong Zhou, Jiahao Shen, Yijian Yang

**Affiliations:** Department of Sports Science and Physical Education, The Chinese University of Hong Kong, N.T. Hong Kong SAR, China; Department of Sports Science and Physical Education, The Chinese University of Hong Kong, N.T. Hong Kong SAR, China; Hinda and Arthur Marcus Institute for Aging Research, Hebrew SeniorLife, Roslindale, Massachusetts, USA; Beth Israel Deaconess Medical Center, Harvard Medical School, Boston, Massachusetts, USA; Department of Sports Science and Physical Education, The Chinese University of Hong Kong, N.T. Hong Kong SAR, China; Department of Sports Science and Physical Education, The Chinese University of Hong Kong, N.T. Hong Kong SAR, China; The Jockey Club Institute of Ageing, The Chinese University of Hong Kong, N.T. Hong Kong SAR, China

**Keywords:** Cognitive, Falls, Gait, Stability, Variability

## Abstract

**Background and Objectives:**

Falls are a major public health concern among older adults, often leading to injuries, impaired mobility, and loss of independence. Dual-task walking, where a secondary task is performed while walking, simulates real-life challenges and is linked to fall risk. This study aimed to investigate how dual-tasking affects stepping strategies, inter-joint coordination, and coordination variability during walking in older adults with and without a history of falls.

**Research Design and Methods:**

Twenty community-dwelling older adults (10 fallers, 10 non-fallers), aged 65 and older, completed a 2-min walking test under three conditions: single-task (ST) walking, motoric dual-task (MDT) walking (holding a glass of water), and cognitive dual-task (CDT) walking (serial subtractions). Gait data were collected using inertial measurement units. Stepping strategies were quantified by the changes in cadence and stride length, while inter-joint coordination was analyzed using vector coding. Two-way repeated measures ANOVA was used to assess task and group effects on variables.

**Results:**

Task-specific adaptations were observed: MDT prompted greater stride length adjustments, while CDT led to more balanced cadence and stride length adjustments (*F* = 8.346, *p* = .010, *η*^2^p = .317). Fallers exhibited more anti-phase coordination in hip flexion–knee flexion than non-fallers during dual-task conditions (*p* ≤ .042). In CDT walking, fallers showed a lower frequency of distal phase in hip flexion–knee flexion and a higher frequency of anti-phase in hip flexion–ankle dorsiflexion compared to ST (*p* ≤ .044). Coordination variability decreased during MDT for hip flexion–ankle dorsiflexion in both groups compared to ST (*p* ≤ .027).

**Discussion and Implications:**

This study provided better understanding on the differences of stepping strategies and phase-specific coordination patterns between older adult fallers and non-fallers, particularly under dual-task walking conditions. The conservative motor control strategies in fallers suggest a prioritization of stability over adaptability, potentially increasing fall risk during complex walking tasks.

**Translational Significance:** Falls among older adults are a major concern due to their impact on mobility and independence. This study reveals that dual-tasking affects stepping strategies and inter-joint coordination differently in fallers and non-fallers, with fallers exhibiting more conservative movement patterns. These findings highlight the importance of considering dual-tasking when assessing fall risk in older adults. By understanding the impact of cognitive and motor tasks on gait, this research can inform targeted interventions to enhance mobility and reduce fall risk, improving both individual quality of life and broader public health outcomes for older adults.

## Background and Objectives

Falls among older adults are a major public health concern due to their association with injuries, reduced mobility, and loss of independence (Bergen et al., 2016). Over 40% of falls occur during walking (Kelsey et al., 2012); therefore, understanding biomechanical differences in gait patterns between older adults with and without a history of falls may provide valuable insights for developing interventions to reduce fall risk (Hamacher et al., 2019; Menz et al., 2003). The intact ability to walk while performing a secondary task, such as talking, carrying an object, or performing a cognitive task (i.e., dual-task walking), which mirrors real-life situations where individuals often engage in multiple activities while walking, is particularly important to maintain balance in everyday activities (Li et al., 2001; Lindenberger et al., 2000). In older adults, such dual-tasking capacity when walking is diminished, and such decline has been closely linked to an increased risk of falls in this population (Montero-Odasso et al., 2012; Zukowski et al., 2021). Therefore, it is important to better understand how dual-tasking interferes with the regulation of gait, which ultimately helps the design of appropriate protocols for fall prevention programs in older adults, especially in those with a high risk of falls (e.g., older fallers) (Ambrose et al., 2013; Li et al., 2001; Lindenberger et al., 2000).

To maintain stability during walking, older adults often adopt conservative gait patterns, such as shorter or more variable step lengths (Hausdorff et al., 2001; Menz et al., 2003). Stepping/speeding strategies, which include adjustments in cadence and stride length, play a critical role in balance and stability during gait (Ardestani et al., 2016). These strategies are classified as neutral, cadence-dominant, or stride length-dominant patterns (Ardestani et al., 2016; Peoples et al., 2024). Neutral stepping involves balanced adjustments in both cadence and stride length, while cadence-dominant and stride length-dominant strategies prioritize either cadence or stride length, respectively (Ardestani et al., 2016; Peoples et al., 2024). Older adults at increased fall risk (e.g., with a history of falls (Deandrea et al., 2010)) may demonstrate distinct preferences or limitations in strategy selection, especially under dual-task conditions, which heighten both motor and cognitive demands (Hollman et al., 2007).

In addition to traditional linear spatiotemporal gait parameters, which have long been associated with fall risk in older adults (Marques et al., 2017; Svoboda et al., 2017), more dynamic aspects of gait, such as inter-joint coordination and coordination variability (CoordV), provide valuable insights into the adaptability and flexibility of movement strategies (Abbasi et al., 2020; Dewolf et al., 2019; Hamill et al., 1999, 2012; Sadeghi et al., 2021). While stepping strategies focus on how cadence and stride length are adjusted for balance, CoordV captures how well the individual adapts their joint movement to meet the demands of walking, particularly under cognitive and motor loads (De Villa et al., 2019; Dewolf et al., 2019). Disruptions in this adaptability, such as lower CoordV, can indicate impaired joint coordination and reduced flexibility in adjusting movement patterns to maintain stability, which is associated with increased fall risk (De Villa et al., 2019; Sadeghi et al., 2021). Therefore, examining both stepping strategies and inter-joint coordination and CoordV—especially in the lower extremities—provides a more comprehensive understanding of gait stability, helping to identify those at higher risk for falls and inform targeted interventions for older adults.

Effective inter-joint coordination, particularly between the knee and ankle, is essential for stability during walking (Hafer & Boyer, 2018). Disruptions in coordination between these joints, such as reduced coupling variability, are associated with a higher risk of falls, as individuals with a history of falls often show lower CoordV compared to non-fallers (Hafer & Boyer, 2018; Sadeghi et al., 2021). When dual-task conditions are introduced, the increased cognitive and/or motor demands can further interfere with this coordination, exacerbating the risk of coordination errors and increasing fall risk (Chiu & Chou, 2013). Metrics such as continuous relative phase and vector coding are commonly used to assess coordination patterns and variability. Continuous relative phase considers both position and velocity, while vector coding focuses solely on position, facilitating clinical interpretation of movement organization and variability (Hamill et al., 1999; Sadeghi et al., 2021).

The present study investigated the impact of dual-tasking on stepping strategies, inter-joint coordination patterns, and CoordV during walking in older adults with and without a history of falls. Using inertial measurement units (IMUs) for naturalistic gait assessment, we aimed to provide actionable insights for fall prevention. We hypothesized that dual-task conditions would elicit distinct stepping strategies and inter-joint coordination patterns, along with lower inter-joint CoordV compared to single-task (ST) walking, with these effects being more pronounced in fallers. By examining these variables across different dual-task conditions, this study seeks to fill critical gaps in understanding the multidimensional coordination challenges associated with fall risk and contribute to the development of effective, tailored fall prevention strategies for older adults.

## Research Design and Methods

### Participants

Twenty community-dwelling older adults (10 fallers and 10 non-fallers), aged 65 and older, participated in this study. Fallers were defined as individuals who reported at least one fall in the past 12 months, with a fall operationally defined as “an unexpected event in which the participant comes to rest on the ground, floor, or lower level” (Lamb et al., 2005). Fallers and non-fallers were matched by age, sex, and body mass index (Table 1). Additional inclusion criteria included: (a) the ability to walk continuously for 10 min without an assistive device; and (b) the capacity to understand test instructions. Exclusion criteria were based on the presence of conditions that could interfere with the testing, including the presence of severe cardiac, pulmonary, or musculoskeletal disorders, gait-affecting pain, significant hearing or vision impairments, and pre-existing neurological disorders (e.g., Parkinson’s disease). The study adhered to the Declaration of Helsinki and was approved by the Institutional Ethics Committee (CUHK SBRE-23-0404B). All participants provided written informed consent before testing commenced.

**Table 1.** Demographic Characteristics of Participants

Variables	Total (*N* = 20)	Fallers (*n* = 10)	Non-fallers (*n* = 10)	*p*
Age (years)	68 (3)	68 (3)	68 (3)	.699
Sex (male/female)	9/11	4/6	5/5	1.000
Body height (cm)	163.90 (9.73)	163.60 (9.47)	164.20 (10.50)	.895
Body weight (kg)	61.80 (10.91)	63.12 (11.82)	60.48 (10.38)	.602
Body mass index (kg/m^2^)	22.93 (2.97)	23.48 (3.18)	22.39 (2.81)	.427

*Notes*: Independent sample *t* tests for normally distributed continuous variables, Mann–Whitney *U* tests for non-normally distributed continuous variables, and the chi-square test for categorical variables.

Sample size was determined using G*Power 3.1.9.6 based on a prior study investigating gait parameters in older adults under different walking tasks using wearable sensors (Soulard et al., 2021). The calculation assumed a power of 80% (1 − *β* = 0.80), an alpha level (type I error) of 0.05, and a medium effect size of 0.30, resulting in a required total sample size of 20 participants.

### Experimental Procedure

**Figure 1.**
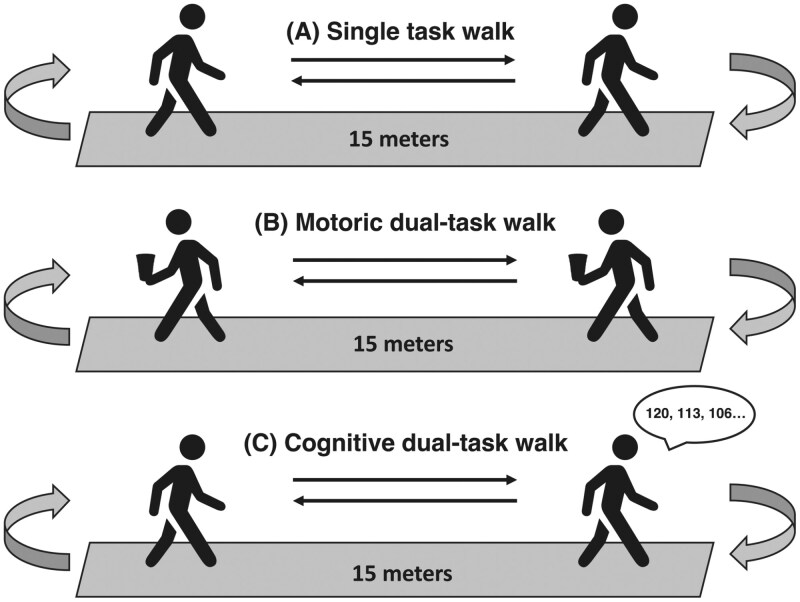
Representative images of gait assessment conditions. *Notes*: (A) usual walk at self-selected pace, (B) motoric dual-task walk while holding a full cup of water, and (C) cognitive dual-task walk while performing a calculation task.

Participants performed a 2-min walking test under the following three conditions (Figure 1):

1. ST: Walking at a self-selected, comfortable speed.
2. Motoric dual-task (MDT): Walking at a self-selected speed while holding a glass of water in the right hand without spilling.
3. Cognitive dual-task (CDT): Walking at a self-selected speed while performing a concurrent serial subtraction task (subtracting 7 from a given number).

For each condition, participants began by standing quietly at the start of a 15-meter walkway marked with bright tape and cones. Upon a verbal countdown (“3, 2, 1, go”), they walked 15-meter, turned 180°, and returned to the starting line, repeating the sequence for 2-min. A 2-min rest was provided between conditions. Testing commenced with the least challenging condition (ST), while MDT and CDT conditions were randomized to minimize order effects.

### Data Processing and Analysis

Kinematic data were collected using wireless IMUs and processed through an integrated software platform, MyoResearch 3.20 (Noraxon USA Inc., Scottsdale, AZ), at a sampling frequency of 200 Hz. Seven IMUs (4.45 cm × 3.30 cm × 1.22 cm) were placed according to the Noraxon standard protocol on the following locations: midpoint of the posterior superior iliac spine, front of the quadriceps (bilaterally), shanks (front on the tibia, bilaterally), and the tops of the feet just below the ankle (bilaterally) (NORAXON, 2024). An initial walking calibration trial was conducted prior to the movement trials.

A 3D motion capture system (Noraxon Ultium Motion) was used to measure lower-extremity kinematics, with joint angles calculated using MyoResearch 3.20. The kinematic data were defined using right-handed Cartesian coordinate systems, where the *x*-axis points along the IMU length, the *y*-axis points to the left of the IMU, and the *z*-axis points outward perpendicular to the IMU surface (NORAXON, 2024). Joint angle decomposition followed the International Society of Biomechanics (ISB) recommendations (Wu et al., 2002), and data were filtered using a robust Kalman filter optimized for IMU-based measurements (Berner et al., 2020). Continuous variables were normalized to the gait cycle (0%–100%) for comparison. Right leg spatiotemporal gait parameters, including cadence and stride length, were computed as averages over 50 gait cycles per trial, excluding the first two cycles. Kinematic data were then exported to MATLAB (Version R2023b, MathWorks, Inc., Natick, MA) for further analysis. Considering previous validation studies demonstrating good-to-excellent validity and reliability for lower-limb joint angles derived from IMUs in the sagittal plane, but raised concerns for measurements in other planes (Kobsar et al., 2020), only sagittal plane kinematics for the hip, knee, and ankle joints were calculated. To evaluate the impact of motoric and cognitive interference on gait variables, the dual-task cost for gait parameters was calculated as shown in Equation (1), a more negative dual-task cost value indicates greater interference from the additional task on gait performance (Goh et al., 2021):


$$\begin{array}{lll} \; & Dual\text{-}task\;cost\;\left(\%\;\right)=\;\;\;\; & \frac{Dual\text{\textasciitilde{}}\text{-}task\;value\;--\;Single\text{-}task\;value}{Single\text{\textasciitilde{}}\text{-}task\;value}\;\times \;100\;\%\; \end{array}$$ (1)

### Stepping Strategy

Stepping strategy was evaluated by analyzing the ratio of relative changes in cadence and stride length between two conditions (e.g., DT vs. ST) (Ardestani et al., 2016). A ratio greater than 1 indicates a cadence-dominant strategy, less than 1 indicates a stride length-dominant strategy, and a ratio of approximately 1 represents a neutral strategy, where both cadence and stride length contribute equally (Ardestani et al., 2016). Stepping strategies for two walking comparisons: MDT versus ST (walking comparison 1) and CDT versus ST (walking comparison 2) were calculated using the following modified equation (Equation 2):


$$\begin{array}{l} Stepping\;\;\;strategy\;\;\;ratio\;\;\;=\;\;\;\frac{\frac{\bar{{Cadence}_{i}^{DT}}\text{-}\bar{{Cadence}_{i}^{ST}}}{\bar{{Cadence}_{i}^{ST}}}}{\frac{\bar{Stride\;\;{\;length}_{i}^{DT}}\text{-}\bar{Stride\;\;{\;length}_{i}^{ST}}}{\bar{Stride\;\;{\;length}_{i}^{ST}}}} \end{array}$$ (2)

Where *i* represents the participant number, DT refers to the dual-task condition, and ST denotes the single-task condition.

### Lower Extremity Inter-Joint Coordination and Coordination Variability

Inter-joint coordination and its variability were quantified using a modified vector coding technique (Needham et al., 2015). Vector coding calculates the coupling angle (*γ*) between joint pairs, representing their coordination pattern, while the standard deviation (*SD*) of this angle at each gait cycle point represents CoordV (van Emmerik et al., 2016). Coordination patterns were categorized as in-phase (movement in the same direction with similar speed), anti-phase (movement in opposite directions with similar speed), proximal phase (predominantly proximal joint movement), and distal phase (predominantly distal joint movement) (Needham et al., 2015) (see Supplementary Figure 1). In this study, joint coupling pairs were analyzed in the sagittal plane, including hip flexion–knee flexion, hip flexion–ankle dorsiflexion, and knee flexion–ankle dorsiflexion, due to their relevance in assessing gait stability.

### Statistical Analysis

Statistical analyses were performed using SPSS (Version 29.0, SPSS Inc., Chicago, IL) and MATLAB (Version R2023b, MathWorks, Inc., Natick, MA). Statistical significance was set at *α* <0.05 for all analyses. Continuous variables were presented as mean and *SD*, and categorical variables were represented as frequency and percentage. Normality was checked by the Shapiro–Wilk test. Two-way (3 × 2) repeated-measures analysis of variance (ANOVA) was conducted to evaluate the effects of task (within-subject factor: ST, MDT, and CDT) and group (between-subject factor: fallers and non-fallers) on gait spatiotemporal parameters, lower-extremity inter-joint coordination pattern frequencies, and CoordV. For dual-task cost and stepping strategy, a separate two-way (2 × 2) repeated-measures ANOVA was conducted with walking comparison as the within-subject factor and group as the between-subject factor. Post hoc comparisons were performed using the Bonferroni method. Partial *η*² values were reported for effect sizes for ANOVA and Cohen’s *d* for post hoc *t* tests. Additionally, one-dimensional statistical parametric mapping (1D-SPM) two-way repeated-measures ANOVA was applied to analyze coupling angle waveforms across the gait cycle, using the open-source spm1d MATLAB code (www.spm1d.org) (Pataky et al., 2013). Adjusted *p* values were used for post hoc paired *t* tests.

## Results

### Gait Spatiotemporal Parameters

Significant task effects were observed in spatiotemporal parameters (Table 2), with post hoc analysis indicated that, for fallers, dual-task conditions resulted in smaller cadence and stride length compared to ST (*p* ≤ .014), dual-task cost for cadence was greater in CDT compared to MDT (*p* = .017); for non-fallers, dual-task conditions resulted in smaller stride length compared to ST (*p* ≤ .006).

**Table 2.** Effects of Group and Task Conditions on Gait and Coordination Variables

Variables	Interaction			Group			Task		
	*F* (*df*)	*p*	*η* ^2^p	*F* (*df*)	*p*	*η* ^*^2^*^ *p*	F (*df*)	*p*	*η* ^2^p
Cadence (steps/min)	2.101 (2)	.137	0.105	0.079 (1)	.781	0.004	15.758 (2)	**<.001**	0.467
Cadence dual-task cost (%)	1.659 (1)	.214	0.084	2.181 (1)	.157	0.108	5.964 (1)	**.025**	0.249
Stride length (cm)	1.282 (2)	.290	0.066	0.930 (1)	.348	0.049	29.152 (2)	**<.001**	0.618
Stride length dual-task cost (%)	1.716 (1)	.207	0.087	0.537 (1)	.473	0.029	0.158 (1)	.696	0.009
Stepping strategy	0.128 (1)	.724	0.007	0.009 (1)	.924	0.001	8.346 (1)	**.010**	0.317
Hip flexion–knee flexion									
In-phase (% of gait cycle)	0.815 (2)	.451	0.043	0.358 (1)	.557	0.020	0.616 (2)	.545	0.033
Anti-phase (% of gait cycle)	1.061 (2)	.357	0.056	6.012 (1)	**.025**	0.250	0.619 (2)	.544	0.033
Proximal phase (% of gait cycle)	0.766 (2)	.472	0.041	1.111 (1)	.306	0.058	1.285 (2)	.289	0.067
Distal phase (% of gait cycle)	0.740 (2)	.484	0.040	0.161 (1)	.693	0.009	8.633 (2)	**<.001**	0.324
Coordination variability (°)	0.107 (2)	.899	0.006	1.838 (1)	.192	0.093	6.981 (2)	**.003**	0.279
Hip flexion–ankle dorsiflexion									
In-phase (% of gait cycle)	0.068 (2)	.934	0.004	0.038 (1)	.848	0.002	4.334 (2)	**.021**	0.194
Anti-phase (% of gait cycle)	0.155 (2)	.857	0.009	0.035 (1)	.854	0.002	5.440 (2)	**.009**	0.232
Proximal phase (% of gait cycle)	0.459 (2)	.635	0.025	0.088 (1)	.770	0.005	1.408 (2)	.258	0.073
Distal phase (% of gait cycle)	1.363 (2)	.269	0.070	1.360 (1)	.259	0.070	1.721 (2)	.193	0.087
Coordination variability (°)	0.107 (2)	.899	0.006	2.363 (1)	.142	0.116	9.822 (2)	**<.001**	0.353
Knee flexion–ankle dorsiflexion									
In-phase (% of gait cycle)	0.598 (1.206)	.478	0.032	2.138 (1)	.161	0.106	1.925 (1.206)	.179	0.097
Anti-phase (% of gait cycle)	0.350 (2)	.707	0.019	0.340 (1)	.567	0.019	0.699 (2)	.504	0.037
Proximal phase (% of gait cycle)	0.302 (2)	.741	0.017	0.484 (1)	.496	0.026	1.374 (2)	.266	0.071
Distal phase (% of gait cycle)	0.387 (2)	.682	0.021	0.574 (1)	.458	0.031	0.889 (2)	.420	0.047
Coordination variability (°)	0.395 (2)	.676	0.021	0.014 (1)	.906	0.001	7.344 (2)	**.002**	0.290

### Stepping Strategy

The two-way repeated-measures ANOVA revealed a significant effect of walking comparison on stepping strategy (*F* = 8.346, *p* = .010, *η*^2^p = .317) (Table 2), with post hoc analysis indicated that MDT was associated with greater stride length adjustments (Mean [*SD*] = 0.55 [0.45]), while CDT was associated with balanced cadence and stride length adjustments (Mean [*SD*] = 1.04 [0.49]) among fallers (*p* = .034).

### Lower Extremity Inter-Joint Coordination Pattern and Coordination Variability

**Figure 2.**
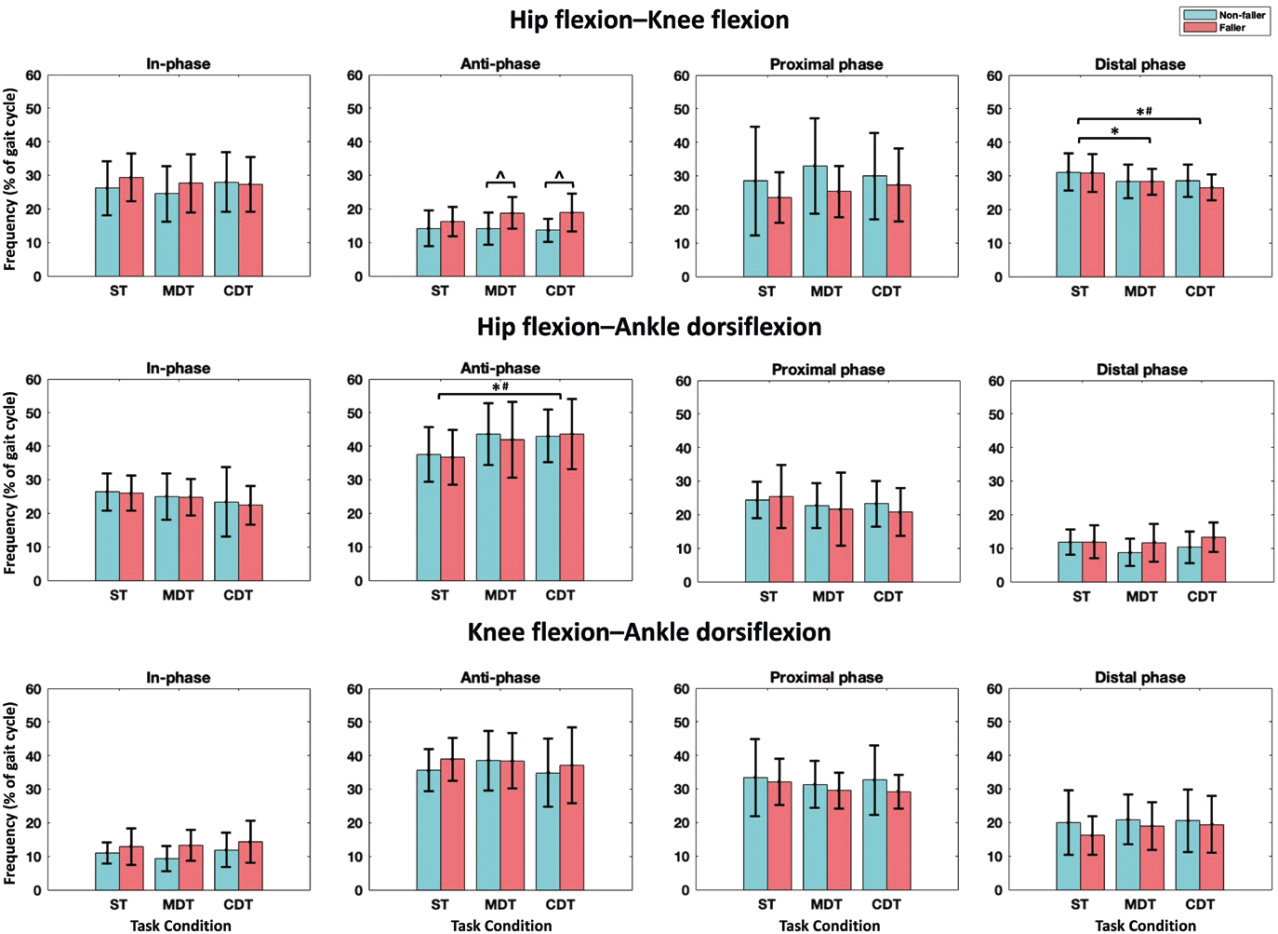
Coordination patterns in fallers and non-fallers across task conditions. *Notes*: CDT = cognitive dual-task; MDT = motoric dual-task; ST = single-task. Error bars represent standard deviations. * indicates significant differences between tasks; ^ indicates significant differences between groups; # indicates significant differences between tasks in fallers (*p* < .05).

No significant interaction effects between group and task conditions were observed for coordination patterns or CoordV parameters (*p* ≥ .269). However, a significant group effect was found for anti-phase coordination of hip flexion–knee flexion (*F* = 6.012, *p* = .025, *η*^2^p = .250), with post hoc analysis indicating that fallers exhibited a higher frequency of anti-phase coordination compared to non-fallers during both the MDT (*p* = .042) and CDT (*p* = .020) (Table 2 and Figure 2). Significant task effects were observed, with post hoc analysis revealing that CDT showed a significantly lower frequency of the distal phase for hip flexion–knee flexion (*p* = .007) and a significantly greater frequency of anti-phase for hip flexion–ankle dorsiflexion (*p* = .044) compared to ST among fallers (Table 2 and Figure 2).

Significant task effects were also observed in CoordV, with post hoc analysis indicated that CoordV for hip flexion–ankle dorsiflexion was smaller during MDT compared to ST among fallers (*p* = .027) and non-fallers (*p* = .013) (Table 2 and Supplementary Figure 2).

**Figure 3.**
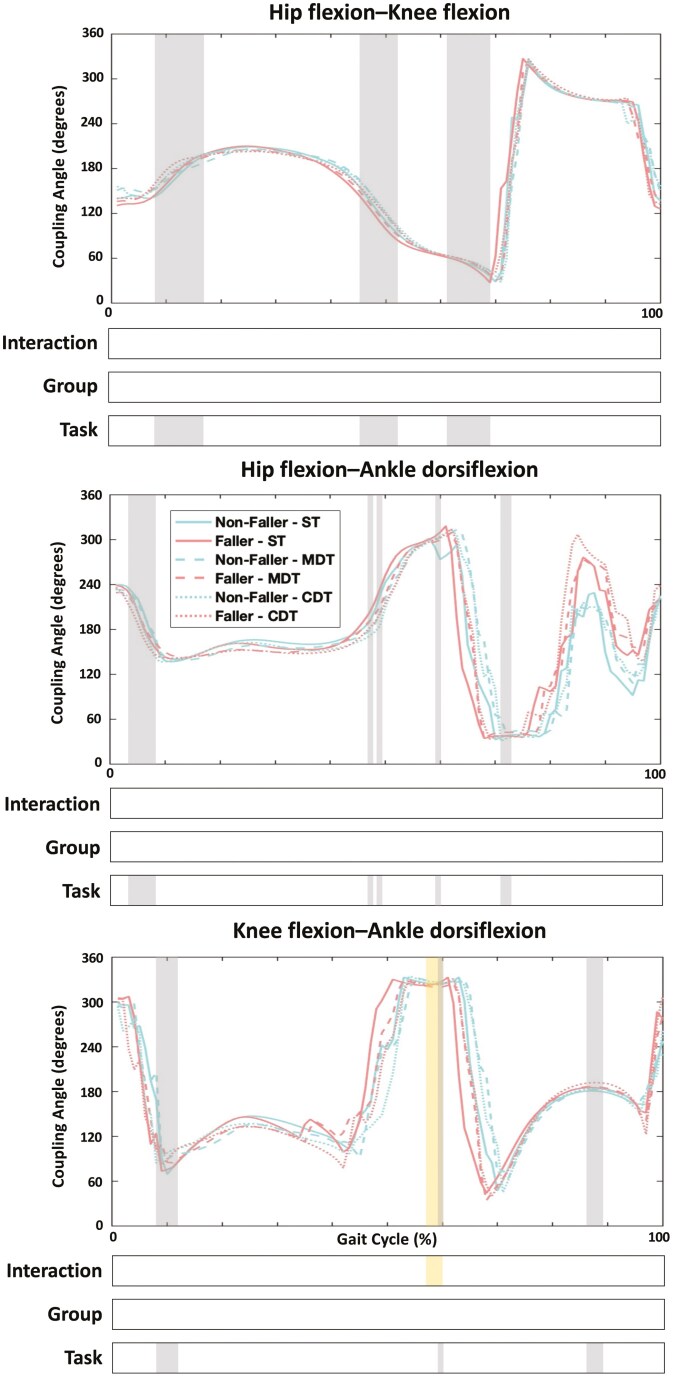
SPM analyses of coupling angles for hip, knee, and ankle joints throughout the gait cycle. *Notes*: CDT = cognitive dual-task; MDT = motoric dual-task; ST = single-task. Significant interaction effects are highlighted in yellow areas; significant task effects are highlighted in gray areas (*p* < .05).

SPM analyses showed a significant interaction effect for knee flexion–ankle dorsiflexion coupling angle waveform during the pre-swing phase (58%–60%) (*F* = 8.944, *p* = .006). Significant task effects were observed during key phases of the gait cycle: for hip flexion–knee flexion: heel strike and loading response (9%–17%), terminal stance and pre-swing (46%–52%), and toe-off (62%–69%) (*F* = 8.449, *p* < .001); for hip flexion–ankle dorsiflexion: heel strike (4%–8%), terminal stance (47% and 49%), pre-swing (60%), and toe-off (72%–73%) (*F* = 8.805, *p* ≤ .035); and for knee flexion–ankle dorsiflexion: heel strike (9%–12%), pre-swing (60%), and terminal swing (87%–89%) (*F* = 8.944, *p* ≤ .036; Figure 3). Post hoc analysis revealed significant differences between MDT and CDT during heel strike (5%–7%) for hip flexion–ankle dorsiflexion among fallers (*t* = 4.781, *p* = .001).

## Discussion and Implications

Our results reveal that fallers exhibited distinct gait adaptations under dual-task conditions, partially supporting our hypotheses. Specifically, fallers demonstrated a greater CDT cost for cadence, reflecting challenges in motor adaptability under cognitive load. Stepping strategy analysis indicated that fallers relied more on stride length adjustments during MDT and more balanced cadence and stride length adjustments during CDT, whereas non-fallers displayed similar task-specific adaptations. Although no significant interaction effects were observed for inter-joint coordination patterns or CoordV, post hoc analyses indicated that fallers showed a higher frequency of anti-phase coordination in hip flexion–knee flexion compared to non-fallers during both MDT and CDT. Fallers also exhibited altered coordination patterns, including a lower frequency of distal phase coordination for hip flexion–knee flexion and a higher frequency of anti-phase coordination for hip flexion–ankle dorsiflexion during CDT. Additionally, CoordV was reduced during MDT for hip flexion–ankle dorsiflexion in both groups, indicating a shift toward a more conservative motor control strategy. SPM analysis further revealed significant task effects on coupling angle waveforms during critical gait phases, such as heel strike, pre-swing, and toe-off, with notable differences between dual-task conditions in fallers.

The greater dual-task cost for cadence observed in fallers during CDT underscores the challenges of performing cognitively demanding tasks while walking. This aligns with prior studies showing that cognitive load interferes with gait mechanics and motor control, likely due to shared neural pathways for cognitive and motor functions (Al-Yahya et al., 2011; Gazzaley & D’Esposito, 2006). The greater dual-task cost in fallers suggests their reduced capacity to adapt body movements when performing cognitively demanding tasks, which may increase the risk of falls in real-life situations where multi-tasking is required (Springer et al., 2006).

The task-specific stepping strategies observed in fallers, particularly their reliance on stride length adjustments during MDT and balanced cadence-stride length adjustments during CDT, suggest that they prioritize spatial stability during MDT and a more balanced approach to both temporal and spatial control during CDT. This finding highlights the adaptability of faller’s gait strategies in response to cognitive load, reflecting their effort to minimize the impact of cognitive distractions on walking performance (Henry & Baudry, 2019; Peterka & Loughlin, 2004). The proportionally greater cadence adjustments during CDT suggest that fallers regulate gait conservatively by adjusting step timing. Temporal adjustments, such as cadence changes, are known to serve as a speed-regulation strategy for older adults facing age-related declines in motor control (Amboni et al., 2013; Ardestani et al., 2016; Peoples et al., 2024). However, excessive reliance on these temporal adjustments may compromise forward propulsion and gait efficiency, further increasing the risk of falls (Amboni et al., 2013; Espy et al., 2010). Adjusting step time brings the center of mass closer to the leading foot, reducing instability and at the expense of energy efficiency (Espy et al., 2010). For fallers, adjusting both cadence and stride length may allow for smaller, more subtle gait adjustments with reduced energy expenditure, facilitating more gradual adaptation to cognitive-motor dual-task conditions (Peoples et al., 2024).

Contrary to previous studies that found significant group differences in inter-joint coordination patterns in the sagittal plane (Sadeghi et al., 2021), our results revealed a group effect only for hip flexion–knee flexion during dual-task walking conditions. However, prior research indicated that fallers exhibit less anti-phase coordination in hip extension–knee flexion compared to non-fallers, which partially supports our results (Sadeghi et al., 2021). The higher knee flexion, along with similar hip flexion across the gait cycle in fallers compared to non-fallers in the current study, may contribute to this finding. Interestingly, these differences were only observed under dual-task conditions, highlighting the sensitivity of dual-task walking to detecting dynamic differences in older adults at high risk for falls (Montero-Odasso et al., 2012; Zukowski et al., 2021). Fallers exhibited a higher frequency of anti-phase coordination for hip flexion–ankle dorsiflexion during CDT, indicating a shift toward opposing joint movements, which may help stabilize the center of mass under cognitive load but at the expense of smooth and efficient movement (Sadeghi et al., 2021). This is consistent with the finding in our study that greater ankle dorsiflexion and smaller hip flexion during the mid-stance and terminal stance phases in CDT may be key contributors to this adaptive strategy. Furthermore, the control adjustment during CDT in the knee joint may primarily drive the differences in distal phase coordination for hip flexion–knee flexion observed in fallers (Al-Yahya et al., 2011). These findings support the notion that while fallers may enhance stability under cognitive load, it comes at the expense of gait fluidity (Sadeghi et al., 2021).

Regarding CoordV, our results showed no significant interaction effects between group and walking task conditions. However, a significant task effect was found, indicating that CoordV was lower during MDT compared to normal walking for hip flexion–ankle dorsiflexion in older adults, regardless of fall history. The reduced CoordV observed in MDT suggests a more rigid motor control approach, potentially limiting older adults’ ability to adapt to dynamic perturbations. This could indicate that the task of holding a glass of water during MDT constrains the movement at the hip and ankle, leading to reduced degrees of freedom. While reduced variability can enhance stability in the short term, it may also reflect constrained motor flexibility, which has been linked to increased fall risk in older adults (Chiu & Chou, 2013; Hamill et al., 2012). Furthermore, when participants were not categorized based on fall history, the results revealed that dual-task conditions caused a reduction in CoordV for both hip flexion–knee flexion and knee flexion–ankle dorsiflexion, particularly between CDT and ST. This result partially supports the notion that lower CoordV may indicate inconsistencies in neuromuscular control and a reduced ability to manage leg motion (Chiu & Chou, 2013; Sadeghi et al., 2021). These findings emphasize the importance of considering both coordination patterns and variability when assessing fall risk and designing interventions, especially under challenging movement tasks.

The SPM results revealed an interaction effect for knee flexion–ankle dorsiflexion coupling at toe-off, with fallers displaying reduced knee flexion and ankle dorsiflexion during CDT. This adjustment suggests a compensatory, more conservative strategy as fallers transit out of the stance phase (Sadeghi et al., 2021), likely to manage the dual cognitive-motor demands, which may impair propulsion and gait fluidity (Montero-Odasso et al., 2012; Muir-Hunter & Wittwer, 2016; Soangra & Lockhart, 2017; Sunderaraman et al., 2019). Additionally, significant task effects were observed for lower extremity inter-joint coupling angle waveforms during critical gait phases, such as heel strike, pre-swing, and toe-off. These phases are essential for maintaining balance and generating forward propulsion (Bulea et al., 2013; Hof, 2008). The significant differences in coupling angles during these phases suggest that dual-task conditions disrupt coordination at moments when precise motor control is crucial (Bayot et al., 2018; Brown et al., 1999). Notably, the differences observed between dual-task conditions during heel strike for hip flexion–ankle dorsiflexion in fallers further emphasize the destabilizing effects of cognitive load on gait coordination in this population (Brown et al., 1999).

In summary, our study provided valuable insights into how dual-tasking influences gait, stepping strategies, and inter-joint coordination patterns in older adults, especially fallers. The use of IMUs for naturalistic gait analysis offers a practical and scalable approach for identifying at-risk individuals and monitoring intervention outcomes (Berner et al., 2020; Porciuncula et al., 2018). While our findings shed light on the biomechanical adaptations related to fall risk, some limitations should be acknowledged. First, the sample size was relatively small, and the participants were relatively young community-dwelling older adults (mean age: 68 years) who are functionally independent, which may limit the generalizability of our findings to frailer or older populations (e.g., individuals aged 80+ years), who often exhibit greater gait impairments and fall risk. Future studies should include broader age ranges and more diverse populations to validate these results. Second, faller status was classified based on self-reported falls over the past year, which may introduce recall bias or underreporting of falls incidence (Garcia et al., 2015). Additionally, the use of IMUs, while advantageous for real-world applications, has shown high validity and reliability for sagittal plane measurements (Kobsar et al., 2020). However, the validity of IMUs for assessing inter-joint coupling angles remains uncertain when compared to optical motion capture systems. Further validation studies are necessary to confirm their accuracy in capturing complex joint interactions and multiplane movements, especially in the context of dynamic, real-word tasks. To increase scalability, future work should focus on developing user-friendly interfaces to translate complex metrics (e.g., inter-joint coordination patterns) into actionable clinical reports. Such advancements will be crucial for establishing IMUs as reliable tools for routine fall risk assessments in clinical practice.

### Clinical Implications

The findings of this study convey significant implications for clinical practice and fall prevention strategies. First, the distinct dual-task adaptations observed in older adult fallers underscore the importance of incorporating dual-task assessments into routine fall risk evaluations. Clinicians and rehabilitation specialists should prioritize evaluating gait performance under both MDT and CDT conditions, as these better reflect real-world challenges than ST assessments alone (Li et al., 2001; Lindenberger et al., 2000; Montero-Odasso et al., 2012; Zukowski et al., 2021). Identifying individuals who exhibit rigid motor adaptations under dual-tasking could help target high-risk populations for early intervention. Second, our results advocate for tailored interventions aimed at improving adaptability in older adults. For fallers, training programs should emphasize tasks that challenge both motor and cognitive domains simultaneously, such as walking while solving problems or carrying objects. Exercises designed to enhance inter-joint CoordV (e.g., obstacle navigation) may help counteract the conservative and stability-focused strategies observed in fallers, thereby improving their capacity to adapt to dynamic environments (Chiu & Chou, 2013; Hamill et al., 2012; Sadeghi et al., 2021). Third, integrating wearable IMUs into clinical practice could enable objective monitoring of gait adaptations during dual-task conditions, allowing clinicians to track progress and adjust interventions based on quantitative feedback (Porciuncula et al., 2018). Finally, our findings highlight the need to move beyond traditional spatiotemporal gait metrics. Assessing coordination patterns and variability during complex tasks provides deeper insights into movement adaptability, which is critical for fall prevention (De Villa et al., 2019; Dewolf et al., 2019; Sadeghi et al., 2021). By adopting a multidimensional approach to gait analysis, practitioners can design more effective, personalized rehabilitation programs that address both stability and flexibility of gait, ultimately enhancing mobility and reducing fall-related morbidity in older adults.

This study reveals distinct gait adaptations in older adults with a history of falls under dual-task conditions, including higher CDT cost for cadence, and balanced adjustments on cadence and stride length during CDT. Fallers also exhibited altered inter-joint coordination patterns and reduced CoordV, reflecting rigid motor control strategies that prioritize stability over adaptability. Wearable IMUs effectively captured these dynamics, highlighting their potential for assessing fall risk among older adults.

## Supplementary Material

igaf055_suppl_Supplementary_Figures_S1-S2

## Data Availability

The study was not preregistered. Please contact the corresponding author for data requests.
