# Supplementary material for: Effects of Dual-Tasking on Stepping Strategy and Inter-Joint Coordination During Walking in Older Fallers and Non-Fallers
Source: Innov Aging. 2025 May 24;9(6):igaf055. doi: 10.1093/geroni/igaf055 (PMC12242380; doi:10.1093/geroni/igaf055)
Supplement: igaf055_suppl_Supplementary_Figures_S1-S2 [file igaf055_suppl_supplementary_figures_s1-s2.docx]

***Innovation in Aging* Supplementary Material: Zeng, Ho, Zhou, Shen, & Yang. Effects of dual-tasking on stepping strategy and inter-joint coordination during walking in older fallers and non-fallers.**


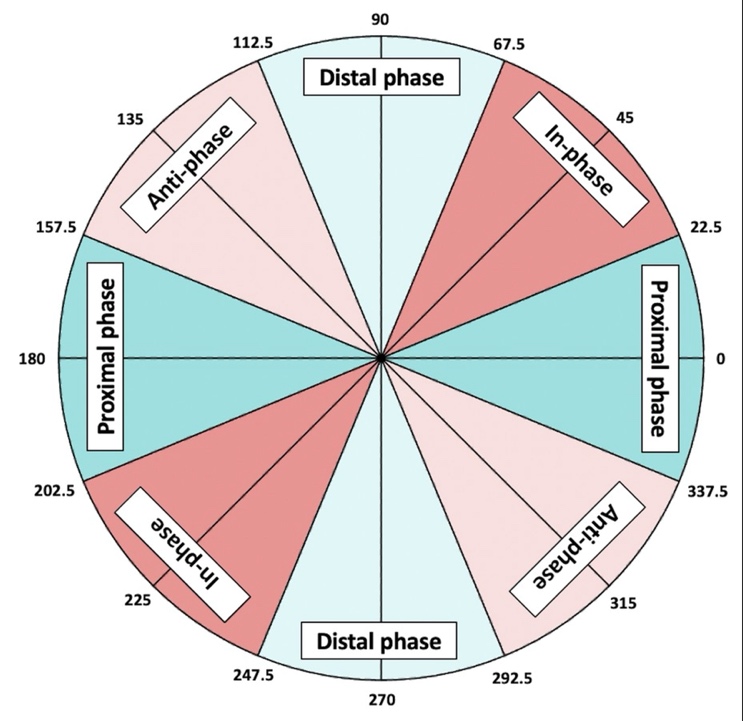


**Supplementary Figure 1**. Definition of coordination patterns according to the coupling angle range.


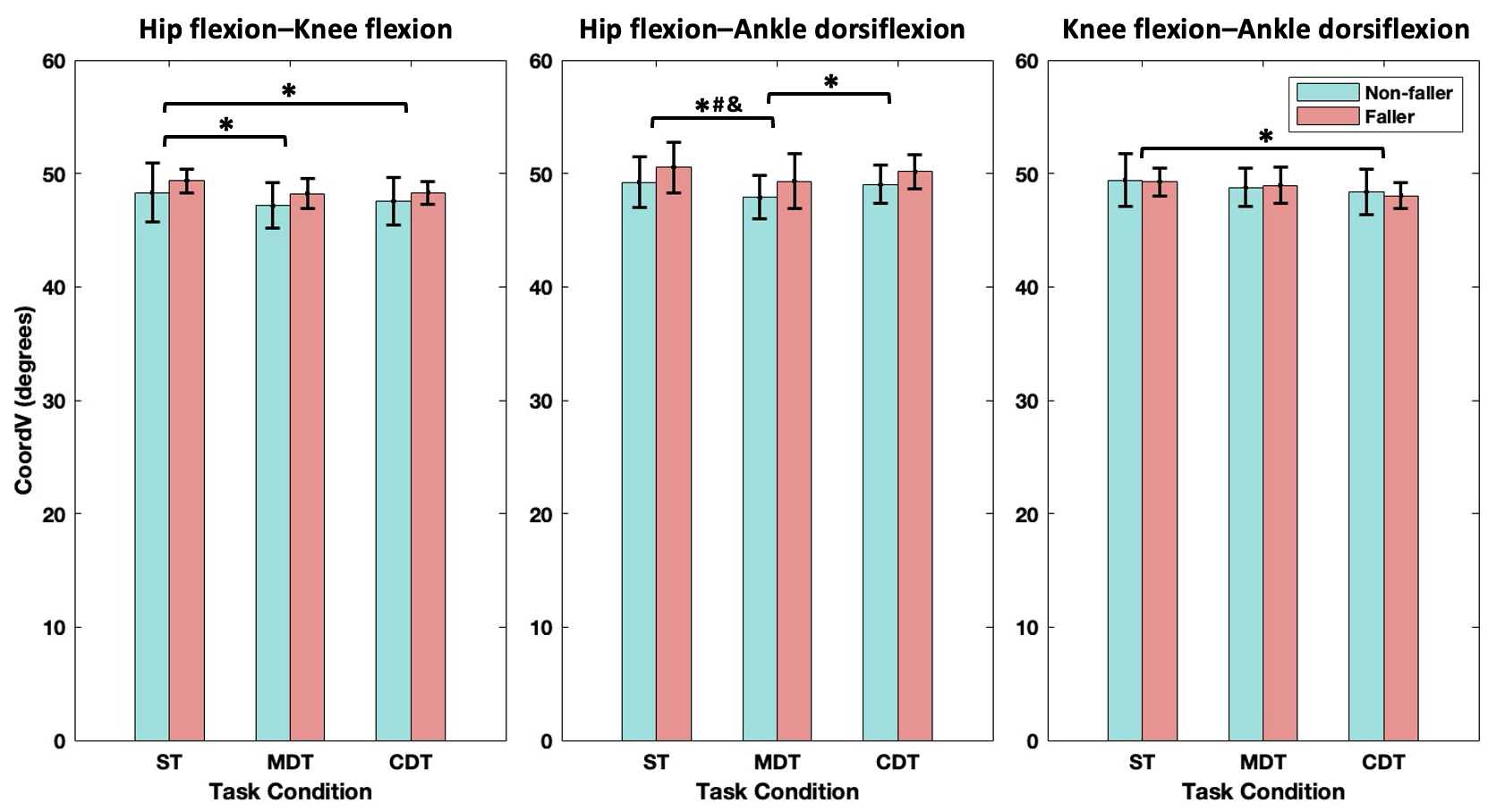


**Supplementary Figure 2**. Coordination variability in fallers and non-fallers across task conditions.

*Notes*: CDT = cognitive dual-task; MDT = motoric dual-task; ST = single-task. Error bars represent standard deviations. * indicates significant differences between tasks; # indicates significant differences between tasks in fallers; & indicates significant differences between tasks in non-fallers (*p* < .05).
